# An Updated Review of Abnormal Hemoglobins in the Turkish Population

**DOI:** 10.4274/TJH.2012.0202

**Published:** 2014-03-05

**Authors:** Nejat Akar

**Affiliations:** 1 TOBB-ETU Hospital, Ankara, Turkey

**Keywords:** Hemoglobin, Variant, Hemoglobinopathy

## TO THE EDITOR

Two previous reviews by Altay and Akar concerning the “Abnormal Hemoglobins in Turkey” appeared in the journal several years ago [1,2]. Since then, several other variants have been reported in both international and national journals. The aim of this mini-review was to compile the newly published abnormal hemoglobins in the Turkish population since these two previous papers [1,2]. 

During the last five years, several variants, each belonging to one family, confirmed with DNA sequencing were reported (Table 1) [3,4,5,6,7,8,9,10,11,12,13,14,15,16,17,18,19,20,21,22]. Two further new variants (Hb İzmir and Hb Edirne) was reported in Turkish population for the first time [18,21]. 

It is interesting that although almost six decades had passed since the first determination of a hemoglobin variant, there are still reports on hemoglobin variants mainly related to clinical and genetic counselling. 

Altay and Akar pointed out that the exact number of subjects having abnormal hemoglobins in Turkish population is not known due to the absence of a national registry system for these conditions [1,2]. So a national registry system collecting clinical and molecular data is needed.

This aim can be achieved under the auspices of the Turkish Hematology Association.

## CONFLICT OF INTEREST STATEMENT

The author of this paper have no conflicts of interest, including specific financial interests, relationships, and/ or affiliations relevant to the subject matter or materials included. 

## Figures and Tables

**Table 1:**
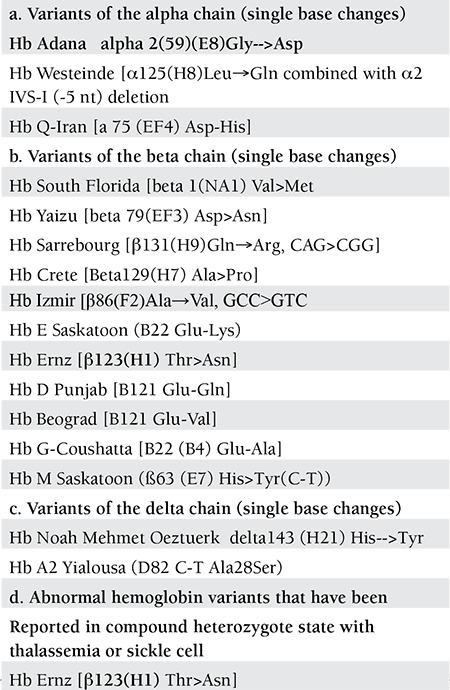
Abnormal hemoglobin variants in the Turkish population published since 2007.

## References

[1] Altay Ç (2002). Abnormal hemoglobins in Turkey. Turk J Hematol.

[2] Akar E, Akar N (2007). A review of abnormal hemoglobins in Turkey. Turk J Hematol.

[3] Atalay A, Koyuncu H, Köseler A, Ozkan A, Atalay EO (2007). Hb Beograd [beta121(GH4)Glu-->Val, GAA-->GTA] in the Turkish population. Hemoglobin.

[4] Atalay EO, Atalay A, Ustel E, Yildiz S, Oztürk O, Köseler A, Bahadir A (2007). Genetic origin of Hb D-Los Angeles [beta121(GH4)Glu-->Gln, GAA-->CAA] according to the beta-globin gene cluster haplotypes. Hemoglobin.

[5] Bissé E, Schaeffer C, Hovasse A, Preisler-Adams S, Epting T, Baumstark M, Van Dorsselaer A, Horst J, Wieland H (2008). Haemoglobin Noah Mehmet Oeztuerk (alpha(2) delta(2)143 (H21)His-->Tyr: A novel delta-chain variant in the 2,3-DPG binding site. J Chromatogr B Analyt Technol Biomed Life Sci.

[6] Koseler A, Bahadır A, Koyuncu H, Atalay A, Atalay AO (2008). First observation of Hb D-Ouled Rabah [beta19(B1)Asn>Lys] in the Turkish population. Turk J Hematol.

[7] Atalay EO, Atalay A, Koyuncu H, Oztürk O, Köseler A, Ozkan A, Demirtepe S (2008). Rare hemoglobin variant Hb Yaizu observed in Turkey. Med Princ Pract.

[8] Kaufmann JO, Phylipsen M, Neven C, Huisman W, Delft P, Bakker-Verweij M, Arkesteijn SG, Harteveld CL, Giordano PC (2010). Hb St Truiden [’68(E17)Asn’His] and Hb Westeinde [’125(H8)Leu’Gln]: two new abnormalities of the α2-globin gene.. Hemoglobin.

[9] Keser I, Yeşilipek A, Canatan D, Lülec G (2010). Abnormal hemoglobins associated with the beta-globin gene in Antalya province, Turkey. Turk J M Sci.

[10] Köseler A, Koyuncu A, Öztürk O, Bahadır A, Demirtepe S, Atalay A (2010). First observation of Hb Tunis [beta124(H2) Pro>Ser] in Turkey. Turk J Hematol.

[11] Curuk MA, Cavusoglu AÇ, Arıcan H, Uzuncan N, Karaca B (2010). Hb Sarrebourg [β131(H9)Gln→Arg, CAG>CGG] in Turkey. Hemoglobin.

[12] Zur B, Hildesheim A, Ludwig M, Stoffel-Wagner BA (2011). First report on Hb Q-Iran in association with alpha-thalassemia in a case of spinal ischemia. Clin Lab.

[13] Arslan C, Kahraman S, Özsan H, Akar N (2011). First observation of hemoglobin Crete [Beta129(H7) Ala>Pro] in the Turkish population |. Turk J Hematol.

[14] Genç A, Çürük MA (2011). Two rare hemoglobin variants in the Çukurova Region of Turkey: Hb E-Saskatoon and Hb G-Coushatta. Turk J Hematol.

[15] Akar N, Arslan Ç, Kürekçi E (2012). First Observation of Hemoglobin M Saskatoon (ß63 (E7) His>Tyr(C-T)) in the Iraqi Population. Turk J Hematol.

[16] Köseler A, Atalay A, Atalay E Ö (2012). HbA2-Yokoshima (delta 25(B7)Gly >Asp) and Hb A2-Yialousa (delta 27(B9)Ala>Ser) in Turkey. Turk J Hematol.

[17] Genc A, Tastemir Korkmaz D, Urhan Kucuk M, Rencuzogullari E, Atakur S, Bayram S, Onderci M, Koc T, Aslan S, Mutalip A, Faruk M, Sevgiler Y, Tuncdemir A (2012). Prevalence of beta-thalassemia trait and abnormal hemoglobins in the province of Adıyaman, Turkey. Pediatr Hematol Oncol.

[18] Celebiler A, Aksoy D, Ocakcı S, Karaca B (2012). A new hemoglobin Variant: Hb Izmir [beta’86(F2)Ala’Val, GCC>GTC; HBB:c. 260C>T. Hemoglobin.

[19] Gunesacar R, Celik MM, Ozturk OH, Celik M, Tümer C, Celik T (2012). Investigation of the clinical and hematological significance of the first observed hemoglobin Ernz variant [β123(H1) Thr>Asn] in the Turkish population. Turk J Med Sci.

[20] Aslanger AD, Akbulut A, Tokgöz G, Türkmen S, Yararbaş K (2013). First Observation of Hb South Florida [beta 1(NA1) Val>Met] in Turkey. Turk J Hematol.

[21] Tabakçıoğlu K, Demir M (2013). Hb-Edirne: A NewdChain Variant: [d53 (D4)Asp> His; HBD c 160G>C].. Hemoglobin.

[22] Durmaz AA, Akin H, Ekmekci AY, Onay H, Durmaz B, Cogulu O, Aydinok Y, Ozkinay F (2009). A severe alpha thalassemia case compound heterozygous for Hb Adana in alpha1 gene and 205 kb double gene deletion.. J Pediatr Hematol Oncol.

